# One Health Relationships Between Human, Animal, and Environmental Microbiomes: A Mini-Review

**DOI:** 10.3389/fpubh.2018.00235

**Published:** 2018-08-30

**Authors:** Pauline Trinh, Jesse R. Zaneveld, Sarah Safranek, Peter M. Rabinowitz

**Affiliations:** ^1^Department of Environmental and Occupational Health Sciences, School of Public Health, University of Washington, Seattle, WA, United States; ^2^Division of Biological Sciences, School of Science, Technology, Education, and Mathematics, University of Washington, Bothell, WA, United States; ^3^Health Sciences Library, University of Washington, Seattle, WA, United States

**Keywords:** one health, microbiome, human, animal, environment

## Abstract

The One Health concept stresses the ecological relationships between human, animal, and environmental health. Much of the One Health literature to date has examined the transfer of pathogens from animals (e.g., emerging zoonoses) and the environment to humans. The recent rapid development of technology to perform high throughput DNA sequencing has expanded this view to include the study of entire microbial communities. Applying the One Health approach to the microbiome allows for consideration of both pathogenic and non-pathogenic microbial transfer between humans, animals, and the environment. We review recent research studies of such transmission, the molecular and statistical methods being used, and the implications of such microbiome relationships for human health. Our review identified evidence that the environmental microbiome as well as the microbiome of animals in close contact can affect both the human microbiome and human health outcomes. Such microbiome transfer can take place in the household as well as the workplace setting. Urbanization of built environments leads to changes in the environmental microbiome which could be a factor in human health. While affected by environmental exposures, the human microbiome also can modulate the response to environmental factors through effects on metabolic and immune function. Better understanding of these microbiome interactions between humans, animals, and the shared environment will require continued development of improved statistical and ecological modeling approaches. Such enhanced understanding could lead to innovative interventions to prevent and manage a variety of human health and disease states.

## Introduction

### The microbiome—from single pathogens to microbial communities

While clinical microbiology has traditionally focused on the role of individual pathogens in human disease, breakthroughs in high throughput DNA sequencing now allow the study of entire microbial communities. The diverse communities of bacteria, archaea, and microbial eukaryotes that compose the human microbiome include non-pathogenic organisms that can impact human health and homeostasis through mechanisms such as nutrient and drug metabolism (1, 2), synthesis of essential vitamins (3), defense against pathogens (4), secondary processing of host bile acids (5), immune modulation (6, 7), resistance and susceptibility against infection (8), and even modification of behavior (9).

### Microbiomes across the one health spectrum

**Figure 1:**
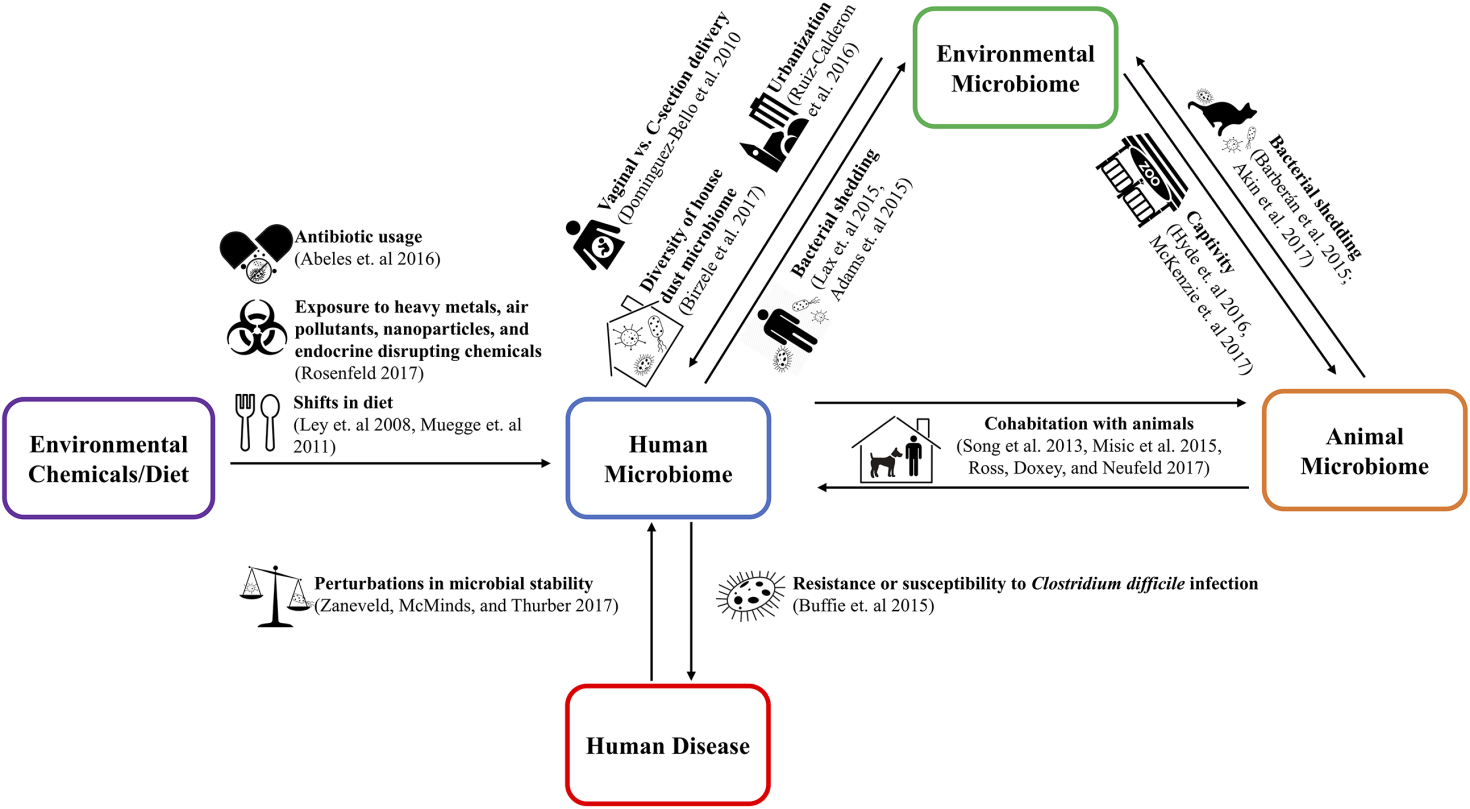
The use of the cutlery, zoo, and city vector icons is licensed under the CC BY 3.0 from www.shareicon.net, www.onlinewebfonts.com/icon, and Thibault Geffroy of the Noun Project, respectively. The dog, pregnancy, balance scale, biohazard, and cat vector icons were taken from Wikimedia Commons and/or have been released into the public domain.

The One Health concept stresses the ecological relationships between human, animal, and environmental health (10). Applying the One Health approach to the microbiome requires examination of both pathogenic and non-pathogenic microbial transfer between humans, animals, and the environment (11–13). These transmission relationships are shown in Figure 1. Understanding the implications of microbiome relationships between the environment and the health of the humans and animals inhabiting it opens the potential for innovative and holistic approaches to diagnosis, treatment, and intervention (14).

Different animal species house unique microbiomes, often of equal or greater complexity compared to the human microbiome. As in humans, animal microbiomes influence the health of livestock, pets, disease vectors, and foundational species that uphold ecosystems. For example, the microbiome of pigs affects their incidence of respiratory disease (15), while the microbiome of reef building corals plays a key role in the response of reef ecosystems to overfishing, nutrient pollution, and global warming (16). Similarly, environments have characteristic microbiomes, and these may impact human health. For example, urbanization parallels increases in allergies, asthma, and other chronic diseases in humans (17), possibly related to reduced exposure to diverse microorganisms (7). Our understanding of environmental microbiomes have increased through sampling efforts in subways (18–21), ambulances (22), restrooms (23), university classrooms and office buildings^1^, as well as outdoor biomes and habitats across the globe (24).

Understanding the human health impact of exposure to microbes from animals or the environment will require integration of exposure assessment frameworks with predictive models of microbial ecology and associated health outcomes. Current evidence suggests that the assembly of the microbiome of humans and other animals is influenced by several processes: exposure to microbes (e.g., from parents, other people, animals, and the environment); filtering of microbial exposures based on the interaction of microbial and host traits (e.g., metabolism and immunity); and the outcome of competitive or cooperative interactions among microbes and phage within the host environment. In this mini-review, we discuss key findings on the interplay between these processes, emphasizing current knowledge of how microbial transfer between humans, animals and the environment may influence human health. We then outline key methodological challenges, and potential solutions, to a predictive understanding of these processes.

## Key aspects of one health microbiome relationships

### Environmental microbiome effects on the human microbiome

The human microbiome can be affected by contact with surfaces, animals (25, 26), and other people; ingestion of food and water; or inhalation (27, 28). Conversely, the gut microbiota may also affect resistance to environmental exposures to parasites and other pathogens by altering innate and adaptive immunity, and through effects on the gut mucosa (29).

The effects of microbial exposure are thought to begin early in life. Vaginal vs. cesarean section (C-section) delivery has been shown to impact microbial seeding of the newborn gastrointestinal tract (30). This change in early life microbial composition carries potential health implications to immune system development influencing health outcomes such as allergic rhinitis, asthma, celiac disease, diabetes mellitus, and gastroenteritis (31).

The built environment can be a source of microbial transfer to inhabitants, and the more closed the environment, the greater potential for such transfer (12, 32). A study of captive Komodo dragons identified microbiome sharing between the enclosure environment and Komodo dragon fecal, salivary, and skin microbiomes (12). The same study found greater beta diversity (differences in species composition) distances between the microbes of humans and their homes than between the Komodo dragons and their enclosures, suggesting that more confined living may be associated with greater built environment effect on host microbiomes (12). Captive vs. wild rearing has been associated with decreases in bacterial community beta diversity in a wide range of animal species (33). For example, placing wild amphibians (34, 35) into enclosed environments diminished the bacterial diversity on their skin unless they were housed with a soil substrate from their natural habitats.

Analogous to captivity in animals, urbanization of human populations results in more enclosed built environments and less diverse diets that could have microbiome effects (36). The household microbiome in rural villages differs from that in more urban settings (36). Similarly, the microbiome of homes of pig farmers have greater microbial diversity and abundance compared to suburban homes (37).

The microbiome of work environments may also affect the human microbiome. In a study of workers in animal research laboratories, SourceTracker (a method for inferring microbial sources) analysis found that the work microbiome contributed to the oral, nasal and skin microbiome of workers (38).

### Microbiome sharing between animals and humans

Elements of microbial communities can transfer between both humans and animals through close contact. A study of the skin microbiota of co-habiting couples found that in addition to person-person microbial sharing, pet ownership was associated with greater skin microbiome diversity (39). Other studies have found microbiome sharing between humans and dogs in the same household, in particular skin microbiota (11). Additionally, cohabiting couples who owned dogs had more skin bacteria in common than other couples without dogs, potentially due to the additional transmission vehicle of a pet (11). Another study found that people living in a household with pets had greater similarities in their nasal and skin microbiomes compared to people who did not have pets, suggesting the influence of pets on promoting microbial exchange (40). Similar patterns have been reported in sharing of microbes between humans and nearby livestock. Pig farming has been found to have significant impact on the nasal microbiome of pig farmers (41). A study of children in Kenyan villages with close livestock contact found that while the greatest amount of gut microbiome similarity was between siblings in the same household, in certain households there was evidence of sharing of microbiome components between children and nearby cows (42).

### Human and animal effects on the environmental microbiome

Source-sink ecological models are based on the concept that there are high quality habitats (sources) in which organisms thrive and lower quality habitats (sinks) into which excess organisms move from the source (13). There is evidence that humans and animals can be a source of microbes moving to their environmental sink. More urbanized and walled-in built environments have a greater content of human-associated microbes compared to more rural and open dwellings (36). Introducing pets into a household can lead to significant changes in the house dust microbiome (43, 44). Often this colonization of environmental sinks by humans and animals is rapid and the environmental microbial signatures transient unless there is continual shedding and presence of the source (13, 45).

### Environmental chemical and pathogen interactions with the human microbiome

Exposure to chemicals in the environment can induce dysbiotic changes to gut microbiome composition or alter the metabolic activity of the gut microbiota (46). Conversely, differences in the composition of the gut microbiota can alter how environmental toxicants are metabolized (46). The health relevance and mechanisms of such interactions are active areas of investigation (47, 48). Taking antibiotics can significantly reduce human gut microbiome diversity and abundance (49). Through enzyme families such as azoreductases, nitroreductases, β-glucuronidases, sulfatases, and β-lyases (46), gut microorganisms can influence xenobiotic metabolism. Gut bacteria can also metabolize a variety of environmental chemicals such as polycyclic aromatic hydrocarbons (PAHs) pesticides, polychlorobiphenyls, benzene derivatives, melamine, artificial sweeteners, and metals (46), altering toxicity (50–52). Other microbiomes beside the gut may modulate response to environmental toxicants. Studies in rodents indicate that the respiratory microbiome, by producing short chain fatty acids, can modulate the airway response to ozone exposure (53).

### Implications of non-pathogenic microbial transfer for human health

While the consequences of transfer of pathogenic microbes are apparent, a growing literature also addresses the health consequences of transmission of non-pathogenic microbes between humans, animals, and the environment. Infants with fewer microbial exposures, such as those experiencing C-section births and urbanized environments, have been found to have higher asthma and allergy risk compared to children growing up on farms, supporting the “hygiene hypothesis” (54). The microbiome of dust samples has been found to differ between houses of children with and without asthma (27), and the diversity of a house dust microbial community can be a predictor of asthmatic status in children residing in that household (28). Gnotobiotic mouse models support the causal effects of non-pathogenic microbial transmission on host health. For example, transfer of feces from obese humans to gnotobiotic mice increases weight gain relative to gnotobiotic mice transplanted with feces from lean humans (55). In combination with the effects of microbiome composition on the metabolism of pharmaceuticals and environmental toxicants, these observations suggest that transfer of non-pathogenic microbes can have significant health consequences.

## Statistical tools for analyzing one health microbiome relationships

### Uncovering interactions between human, animal, and environmental microbiomes

One Health approaches aim to understand the reciprocal influences of human, animal, and environmental microbiomes on one another in order to ultimately design interventions that improve human health. A first step in doing so is to characterize how a factor of interest alters microbial communities and human health. Follow-up studies that directly manipulate the microbiome (e.g., in gnotobiotic mice) can help to test whether observed microbiome changes play a causal role, or represent a secondary consequences of changes in health status.

After sample collection and extraction of microbial DNA, marker gene sequencing amplifies a gene of interest and then sequences it to infer the phylogeny and taxonomic composition of a microbiome. Commonly targeted genes include the 16S ribosomal RNA(16S rRNA) gene for studies of bacteria and archaea, ITS2 for studies of fungi, or the 18S rRNA gene for microbial eukaryotes. In contrast, shotgun metagenomics fragments and sequences all available microbial DNA, and then analyzes the set of sequences to identify not only bacterial species, but also the presence, absence, and variety of particular genes (56, 57). When conducted at very high sequencing depth, shotgun metagenomics can also recover partial microbial genomes using compositional binning or correlations in genes belonging to the same organism across samples (58). Whatever technology is used, careful sampling design that addresses the specific hypotheses to be tested, and collection of sufficient metadata (e.g., on confounding variables) are essential to allowing results to be interpretable (59).

Typical questions thatcan be addressed through microbiome sequencing approaches, in combination with targeted experiments, include: what external factors influence the microbiome; how each of those factors alters microbiome richness and evenness (60–62), composition (63–66), stability, and function; the source (67), direction, and magnitude of microbial transfers; and which microbial species (or species consortia) mediate key health outcomes (Box 1).

Box 1 **Microbial ecology methods.**

Two primary metrics for describing a microbiome are alpha and beta diversity. Alpha diversity examines the number (richness) and distribution (evenness) of taxa within a single population (60). Specific metrics for quantifying richness include the number of observed observational taxonomic units (OTUs), and the Chao1 richness estimator (60, 61). Evenness is typically measured with equitability or the Gini index (which is also used to characterize income inequality in economics). The Simpson index (68) and the Shannon diversity (69) metric incorporate both richness and evenness. Faith's phylogenetic diversity is a richness measure that weights richness according to phylogenetic diversity (70). Whatever method is chosen must account for differences in the numbers of sequences in each DNA library. This is commonly addressed by either randomly resampling each sample to an even number of sequences (rarefaction) or through statistical models designed to incorporate sequencing depth (71).

Beta-diversity metrics examine the differences between two microbiome communities by quantifying the overlap of shared taxa between them (60). Metrics of beta diversity include weighted and unweighted UniFrac, Bray-Curtis dissimilarity, and Jaccard index (60, 61, 72, 73). Beta-diversity metrics can be quantitative (e.g., weighted UniFrac), taking into account sequence abundance, or qualitative (e.g., unweighted UniFrac), considering only the presence or absence of sequences (74). They can also be phylogeny-based (e.g., UniFrac) or not (e.g., Bray-Curtis) (61). Beta diversity metrics can be visualized through PCoA plots and different sample categories, such as cases and controls, tested for differences in composition and dispersion using a variety of methods such as adonis, ANOSIM, PERMANOVA etc. (74). While predictable shifts in beta-diversity associated with disease have been most commonly studied, care should be taken to characterize variance as well as increased microbiome instability is associated with multiple human and animal diseases (75). PERMDISP (76, 77) and the betadisper (78) function in the vegan R package are two common methods for characterizing dispersion in cohort data, whereas time-series datasets allow assessment of the volatility of microbiomes within individuals over time.

The bacterial 16S ribosomal RNA (rRNA) gene contains 9 hypervariable regions (V1–V9) that are surrounded by conserved regions in most bacteria (79). Choice of variable region and primers have been shown to have significant effects on biological conclusions (80). While there is no consensus on the “best” target region, the most commonly sequenced regions surround V2, V4, and V6. V2 and V4 have been found to have the lowest error rates during taxonomic assignment (63, 64) while the V4–V6 regions have the highest phylogenetic resolution (62). The V1–V3 and V1–V4 regions have also been highly recommended for bacterial analysis because these regions provide more reliable estimates of species richness and identification, are more divergent and therefore offer more phylogenetic resolution, and have corresponding sequences that have been cataloged extensively in databases such as RDP (81). Due to the significantly different results that come from selection of target hypervariable regions and equivalently primers, it is important for cross-study comparability to select a standardized region. In the absence of a strong motivation for picking another hypervariable region, there are significant potential advantages to sticking with regions cross-comparable with other reference datasets of interest e.g., Human Microbiome Project or Earth Microbiome Project.

There is also no consensus on the “best” reference database for taxonomic assignment of DNA sequences.This remains an important consideration since the taxonomy used can have significant effects on study results (64). A recent paper by Balvočiute and Huson compared the SILVA (66), RDP (63), Greengenes (65), NCBI (82), and Open Tree of Life Taxonomy (OTT) (83) databases (84). The authors recommended the NCBI taxonomy for studies that use both shotgun and 16S marker gene sequencing data as it is one of the largest taxonomies and is updated daily (84). However, a major advantage of SILVA and Greengenes is that they are tree-based whereas NCBI is just a taxonomy. For researchers interested in eukaryotic organisms, the SILVA taxonomy is a widely used reference as it contains comprehensive information for not only Bacteria and Archaea, but also Eukarya (66). For 16S marker gene sequencing studies that involve environmental samples, SILVA and Greengenes may be of particular utility as these taxonomies contain a number of phylum-level taxa specific to environmental sequences (66). Additionally for 16S sequencing studies, it may be of interest to note that RDP and SILVA are updated more frequently than Greengenes (last updated in August 2013). Ultimately, the choice of reference taxonomy is dependent on the research objective keeping in mind that there are strengths and limitations with each reference database and that choosing a taxonomic reference database similar to reference datasets of interest would be advantageous for cross-study comparability. Of additional note, data resources, including the percentage of microorganisms with available genome sequences, tend to be much lower in non-model animals than in humans. This can limit the accuracy of methods such as predictive functional profiling, which rely on sequenced genomes to estimate functional roles from phylogeny (85) or taxonomy (Tax4Fun), as well as the accuracy of taxonomy assignment methods for shotgun metagenomics that depend on knowledge of gene family combinations that are diagnostic for particular strains (86).

### Toward predictive models of microbial transfer

Predicting and potentially manipulating transfers of microbes between humans, animals, and the environment depends on an understanding of the factors that allow or inhibit transfers. This objective parallels the role of predictive models—such as human exposure modeling—in exposure science, with the added complication that the exposures in question are biological entities with their own complex ecological and evolutionary dynamics.

Indicator species analysis has been a key method for detecting the source of microbial contamination of sites in the environment (87). However, indicator species methods typically assume that there are species unique to source vs. sink samples. Dirchlet multinomial models are an alternative approach that try to explain observed microbial communities as a mixture of microbial profiles from different source communities, as occurs during transfer of microbes. For example, SourceTracker software uses a estimates the proportion of a particular microbiome comprising microbes from a specified source (88). However, because microbial transmission can be circular in a closed system, longitudinal datasets or additional experiments are often needed to establish the direction and dynamics of microbial transmission. Lax et al. addressed this by applying SourceTracker longitudinally to examine transmission of microbes between seven families and their homes during periods of continuous residence vs. moves (45). They additionally coupled their SourceTracker analyses with dynamic Bayesian networks to test the direction of microbial transfer and confirm that humans were more likely to be sources of bacteria than physical surfaces. One limitation of SourceTracker is that all source samples are considered separately—interdependence between source environments is not modeled. One solution currently being explored by packages such as BioMiCo is to model source environments hierarchically (89). All of these methods still assume that transfer was instantaneous. Accurately predicting the consequences of microbiome transfer over time, or detecting microbiome changes due to past transfers will likely require more detailed and environment-specific models that incorporate microbe-microbe, and microbe-phage interactions.

The ecological concept of keystone species predicts that certain species can exert especially large influences on microbial interaction networks (67, 90, 91), including those of the gut microbiome (92, 93). However the complex interactions in ecological systems can make it difficult to identify and validate the presence and role of such keystone species (93). Lotka-Volterra models of predator-prey interactions are one approach to inferring competitive and cooperative interactions within microbial communities (94). The generalized Lotka-Volterra framework models the dynamics of an arbitrary number of species and can be extended using a combination of machine learning and ecological modeling to infer underlying microbial networks and model time-dependent perturbations (94). Data driven approaches such as the generalized Lotka-Volterra framework benefit greatly from time-series data, emphasizing the need for longitudinal studies. Establishing causality rather than just correlation will continue to be a major challenge for One Health-related microbiome research. For example, competitive or cooperative microbial interactions inferred through modeling can be tested experimentally (*in vitro* or in model organisms).

Several recent advances have applied ecological insights to predict the response of microbiomes. For example, an understanding of the role of niche competition in microbial establishment allowed Kearney et al., to engineer a microbe's establishment in the human gut by matching the strains known metabolic capabilities to construction of a niche through addition of seaweed to the diet (95). Predictions of microbial competition or cooperation—based on positive or negative co-occurrence patterns—successfully predicted which microbes would increase alpha-diversity in *Nematostella vectensis* (96). A similar approach has been applied in predicting zoonotic viral infections. Risk factors for the emergence of zoonotic viral infection from mammal hosts include the phylogenetic relatedness of the host to humans, animal taxonomy (a proxy for traits), and range overlap with human populations (likely a proxy for exposure) (97).

### Methodological challenges particular to one health approaches

Because One Health approaches often compare changes in human microbiomes with multiple animal or environmental interactors, several particular methodological challenges arise that may not apply to purely clinical studies.

One such challenge for integrated microbiome studies of humans, animals, and environments will be to disentangle the role of host genetics on microbial transmission dynamics and health impacts. Across human patients, genetic differences have been recognized as an important factors that shapes the gut microbiota (98). Susceptibility to microbial transfer may be related to host genetic factors that influence the presence/absence of microbial strains. Host genetics may also potentially influence the potential health impacts of transfers that do occur. As such, future studies examining the role of host genetics in modulating transmission dynamics are promising avenues for research. However, the role of host genetics is likely much larger when comparing breeds of domesticated animals or across animal species. A recent analysis of zoonotic viral infections found bats to be an especially important vector (97). Yet there are between ~950 and 1,250^2^ species of bats worldwide, each with their own variation in diet, life-history strategy, degree of overlap with human habitation, and extent of intra-specific genetic variation. Therefore, One Health approaches will benefit from ecological and evolutionary studies that distill natural variation in host-microbe interaction into a more tractable number of general rules. For example, many groups of animal and plant groups exhibit phylosymbiosis (99), in which the overall composition of microbial communities between species follows the structure of the phylogenetic tree that relates their hosts.

Animal species are related by the tree-like structure of evolution. In comparing microbiomes between multiple animal species, correcting for phylogenetic dependence using phylogenetic comparative methods is essential (100). Comparing any traits, including microbiome composition, across animal species using traditional, phylogenetically-naïve statistic across species implicitly assumes that all species are equally related (a “star phylogeny”). This can induce very high rates of false positives (100). If two traits happen to arise together once by chance, but the lineages in which they arose subsequently speciate extensively, the traits will appear to be strongly correlated, even if the two traits have no causal bearing on one another at all. This problem is well understood in ecology and evolution, and can be addressed using methods such as phylogenetic independent contrasts and phylogenetic generalized least squares (PGLS). A number of R packages implement these methods, including ape (101), phangorn (102), phytools (103), picante (104), caper (105), Geiger (106), and phylolm (107). Similar considerations apply when comparing features—such as genes underlying host range—across diverse microbial pathogens or symbionts (108).

## Future directions and challenges

In humans, fecal transplants show significant clinical promise for treatment of recurrent *Clostridium difficile* infection (109). An improved understanding of the health consequences of different microbiome configurations in humans and animals could similarly open up opportunities for clinical interventions that modify the microbiome directly, as well as health policy interventions that modify it indirectly. With the growing availability of 'omics data, assessing the health risks and benefits from particular microbiome relationships will require new predictive models that take into account complex microbiome interactions (110). Incorporation of ecological models to predict the behavior of the ecosystem into such risk assessment may increase predictive capability. This could include assessing and modifying the built environment microbiome to enhance the health of humans and animals in the household. Another possibility would be to regulate the microbiomes of companion animals through diet or probiotics to impact their effect on the health of cohabiting humans. Such intervention possibilities remain speculative at this point, but a goal of One Health microbiome research should be to define healthy coexistence and test interventions to optimize microbiome exchange between humans, animals, and the environments they share.

## Author contributions

PT, JZ, and PR wrote and edited various aspects of the manuscript. SS assisted in project conception and search methods. PT and PR crafted the project concept.

### Conflict of interest statement

The authors declare that the research was conducted in the absence of any commercial or financial relationships that could be construed as a potential conflict of interest.

## Footnotes

^1^Sloan Program on the Microbiology of the Built Environment. Available online at: https://www.microbe.net/alfred-p-sloan-foundation.

^2^From the Integrated Taxonomic Information System on-line database. ITIS. Available online at: http://www.itis.gov (Accessed July 7, 2018).
